# Prevalence and Patterns of Antibiotic Prescribing Among Children Aged 1–7 Years in Primary Health Care Centers in Prishtina and Ferizaj, Kosovo (2022–2025): A Retrospective Observational Study

**DOI:** 10.3390/antibiotics14121282

**Published:** 2025-12-18

**Authors:** Fitim Bexhet Alidema, Arieta Hasani Alidema, Lirim Shefki Mustafa, Mirlinda Havolli, Fellenza Abazi

**Affiliations:** 1 Department of Pharmacology, Faculty of Pharmacy, UBT College, 10000 Prishtina, Kosovo; 2 Department of Nursing, Faculty of Medical, UBT College, 10000 Prishtina, Kosovo; 3 Faculty of Health Sciences, University of Maribor, 2000 Maribor, Slovenia

**Keywords:** antibiotics, pediatric prescribing, empirical treatment, primary care, Kosovo, antimicrobial resistance, guideline adherence

## Abstract

**Background:** The inappropriate and empirical use of antibiotics in early childhood remains a major global public health concern, contributing significantly to the rise of antimicrobial resistance (AMR). In Kosovo, the COVID-19 pandemic further influenced prescribing behaviors in primary care, increasing the reliance on symptom-based treatment in the absence of laboratory confirmation and age-appropriate formulations. Aim: This study aimed to assess the prevalence and patterns of antibiotic prescribing among children aged 1–7 years in primary health care centers in Prishtina and Ferizaj from January 2022 to December 2025, and to compare regional differences in prescribing practices and guideline adherence. **Methods:** A retrospective observational study was conducted using data from the national electronic health record system and protocol books. All pediatric visits for children aged 1–7 years with infectious diagnoses were included. Descriptive statistics, Chi-square tests, and multivariable logistic regression were used to evaluate prescribing prevalence, demographic and seasonal variations, and independent predictors of antibiotic use, including assessment of appropriateness based on international pediatric guidelines. **Results:** Of 4320 pediatric visits, 1328 (30.7%) resulted in an antibiotic prescription. Prescribing prevalence was higher in Ferizaj (34.2%) than in Prishtina (28.5%, *p* < 0.01). Amoxicillin–clavulanic acid (42.9%) and amoxicillin (21.5%) were the most frequently prescribed agents, while macrolides (11.7% vs. 6.2%) and cephalosporins (7.9% vs. 3.4%) were more common in Ferizaj. Only 61.4% of prescriptions were fully guideline-concordant. Younger age (1–3 years), winter season, and residence in Ferizaj were independently associated with higher odds of receiving an antibiotic. **Conclusions:** Pediatric antibiotic prescribing in Kosovo remains high and predominantly empirical, reflecting real-world limitations in diagnostic capacity and formulation availability. Significant proportions of partially appropriate and inappropriate prescriptions highlight the need for standardized pediatric guidelines, improved diagnostic support, and strengthened stewardship initiatives within primary care.

## 1. Introduction

Antibiotic overuse and inappropriate prescribing in early childhood have become major global public health concerns, contributing directly to the rise of antimicrobial resistance (AMR). Over the last decade, international surveillance networks such as ECDC, ESAC-Net, and CAESAR have documented persistent and disproportionate outpatient antibiotic consumption among pediatric populations, especially in Southeast Europe, where stewardship systems are less established [1,2,3]. The World Health Organization (WHO) has listed AMR among the top ten global health threats, emphasizing that uncontrolled and empiric prescribing—particularly in children—accelerates bacterial resistance and compromises treatment effectiveness [4].

Children aged 1–7 years are among the most frequently exposed groups to antibiotics. Respiratory tract infections, otitis media, and urinary tract infections represent the leading indications for outpatient prescriptions; however, numerous studies show that a substantial proportion of these treatments deviate from international pediatric guidelines and are often unnecessary or inappropriate [5,6,7,8]. Early-life exposure to antibiotics has been linked to gut microbiota disruption, increased risks of asthma and allergic diseases, and potential long-term metabolic consequences [9,10].

Across Europe, substantial progress has been made through national action plans, prescribing guidelines, point-of-care diagnostic expansion, and stewardship interventions aimed at optimizing pediatric antibiotic use [11,12]. Despite these developments, significant variation persists between countries: Western European nations report declining prescribing rates, while many Eastern and Southeastern European regions continue to demonstrate high empirical use of broad-spectrum antibiotics, including macrolides and cephalosporins [13,14,15].

Kosovo represents one of the countries where national pediatric prescribing guidelines, electronic surveillance systems, and formal stewardship structures in primary care remain limited. Existing local reports indicate heavy reliance on broad-spectrum antibiotics, frequent empirical prescribing without microbiological confirmation, and inconsistent availability of age-appropriate liquid formulations in both outpatient and hospital settings [16,17,18]. These challenges were further amplified during the COVID-19 pandemic, when reduced access to diagnostic testing and heightened parental expectations contributed to increased symptom-based prescribing.

Municipal-level differences also play a notable role. Prishtina, the capital city, has comparatively greater access to pediatric specialists and diagnostic tools, whereas Ferizaj relies more heavily on general practitioners, with limited point-of-care testing capacity (e.g., CRP rapid test, streptococcal antigen test). Understanding these differences is essential for identifying context-specific drivers of prescribing and for informing targeted stewardship interventions. Given this context, this study aims to:

(1) Determine the prevalence and patterns of antibiotic prescribing among children aged 1–7 years in primary health care centers in Prishtina and Ferizaj;
(2) Assess the appropriateness of prescriptions relative to international pediatric guidelines; and
(3) Evaluate demographic, seasonal, and regional predictors of prescribing.

By integrating post-pandemic data from two major municipalities, the study provides one of the first detailed, real-world assessments of pediatric outpatient antibiotic use in Kosovo and generates evidence to support national stewardship, diagnostic strengthening, and pediatric prescribing education initiatives.

## 2. Results

The distribution of prescribed antibiotics by class across the two municipalities is summarized in Table 1, Table 2, Table 3, Table 4, Table 5, Table 6, Table 7, Table 8 and Table 9.

## 3. Discussion

This study provides the first post-pandemic evaluation of pediatric antibiotic prescribing in primary health care settings in Kosovo. The overall prescribing prevalence (30.7%) is comparable to findings from several Southeast European countries but remains higher than rates observed across Western Europe, where strong stewardship structures and national pediatric guidelines are routinely implemented [1,2]. Recent ESAC-Net surveillance reports demonstrate that antibiotic consumption in EU/EEA countries declined steadily from 2013 to 2020, dropped sharply during the COVID-19 pandemic, and subsequently increased again in 2022–2023 as health care utilization normalized [19,20,21,22]. These updated European trends highlight the need for Kosovo to implement standardized pediatric prescribing and monitoring systems.

Broad-spectrum antibiotic use—particularly amoxicillin–clavulanic acid, macrolides, and cephalosporins—was high in both municipalities. Although penicillins remain the dominant outpatient antibiotics in the EU/EEA, recent data show reductions in macrolide (J01F), cephalosporin (J01D), and quinolone (J01M) use across many European countries [23]. In contrast, Kosovo continues to demonstrate elevated macrolide and cephalosporin prescribing, especially in Ferizaj, diverging from current European trajectories. Global consumption patterns also indicate increasing antibiotic use in many low- and middle-income countries between 2016 and 2023, with projections of further growth through 2030 [24].

Younger children (1–3 years) had significantly higher prescribing rates, consistent with international literature attributing increased antibiotic use in toddlers to diagnostic uncertainty, reduced cooperation during clinical assessment, and parental expectations rather than to higher bacterial infection prevalence [6,7].

### 3.1. Diagnostic Uncertainty as a Driver of Pediatric Antibiotic Prescribing

Diagnostic uncertainty represents one of the most important drivers of empirical antibiotic prescribing in pediatric primary care. Young children frequently present with nonspecific clinical symptoms such as fever, cough, nasal discharge, irritability, and gastrointestinal disturbances, which often overlap between viral and bacterial infections. In many cases, clinical examination alone is insufficient to reliably distinguish bacterial from viral etiologies, leading physicians to prescribe antibiotics as a precaution rather than based on confirmed infection [5,6].

In the present study, the significantly higher prescribing rate observed among children aged 1–3 years strongly reflects this diagnostic challenge. At this age, clinical assessment is limited by poor cooperation, incomplete verbal communication, and increased parental anxiety. These factors contribute to a higher perceived risk of missing serious bacterial infections and often result in defensive prescribing practices [6,15].

The problem is further exacerbated by the lack of routine point-of-care diagnostic testing in Kosovo’s primary health care system. Rapid diagnostic tools such as C-reactive protein (CRP) testing, rapid streptococcal antigen detection tests, and urine dipsticks are inconsistently available, particularly in Ferizaj. In the absence of laboratory confirmation, antibiotic prescribing decisions are predominantly guided by clinical judgment rather than microbiological evidence [5,8].

Substantial international evidence demonstrates that the implementation of point-of-care diagnostics significantly reduces unnecessary antibiotic use in pediatric populations, particularly for respiratory tract infections where viral pathogens predominate [5,8]. Countries with established diagnostic pathways report improved guideline adherence, reduced empirical use, and lower exposure rates to broad-spectrum antibiotics. The limited availability of such diagnostic support in Kosovo likely contributes directly to the empirical prescribing patterns observed in this study and underscores the urgent need for diagnostic strengthening within pediatric antimicrobial stewardship programs.

#### Impact on Vulnerable Pediatric Populations

Although the present study focuses on antibiotic prescribing patterns, individual-level data regarding vulnerability status—such as prematurity, immunodeficiency, or chronic medical conditions—were not systematically available in the electronic health record system. Consequently, the exact proportion of vulnerable children within the study cohort could not be quantified.

However, international data suggest that approximately 10–20% of children in primary care settings live with at least one chronic medical condition or immune vulnerability. These groups are disproportionately affected by antimicrobial resistance due to frequent health care contact, increased antibiotic exposure, and greater risk of complicated infections [19,20].

It is noteworthy that children aged 1–3 years, who accounted for the highest antibiotic prescribing rates in the present study, represent an inherently vulnerable population due to immature immune systems and increased susceptibility to severe infection outcomes. Antibiotic exposure at early stages of life is known to disrupt gut microbiota and increase long-term risks related to immune dysregulation and resistance development [11,16].

The absence of stratification by vulnerability status represents an important limitation of the present study and highlights the need for future research that integrates immunological and clinical risk profiling into pediatric antibiotic surveillance systems.

### 3.2. Multidrug Resistance in Pediatric Infections

Multidrug-resistant pathogens represent a rapidly escalating threat to pediatric populations worldwide. Resistant organisms such as methicillin-resistant *Staphylococcus aureus* (MRSA), extended-spectrum beta-lactamase (ESBL)-producing *Enterobacterales*, and macrolide-resistant *Streptococcus pneumoniae* are increasingly reported in both community and hospital settings [19,25].

Although microbiological resistance data were not available in the present study, the high utilization rates of broad-spectrum antibiotics—particularly amoxicillin–clavulanic acid, macrolides, cephalosporins, and metronidazole—raise important concerns regarding resistance selection pressure. Frequent empirical use of cephalosporins is strongly associated with increasing ESBL prevalence, while macrolide overuse has been linked to rising pneumococcal resistance across Europe [19,26]. Similar prescribing patterns among pediatric populations have also been reported in Central and Eastern European countries, including Hungary [22].

European surveillance networks, including ECDC and CAESAR, consistently report macrolide resistance in *Streptococcus pneumoniae* exceeding 25% in several countries from Southeastern Europe, as well as increasing detection of ESBL-producing *Enterobacterales* in pediatric infections [25,26]. These findings underscore the urgent need for national pediatric antimicrobial stewardship strategies aligned with international frameworks, including the WHO Global Action Plan on Antimicrobial Resistance and European antimicrobial resistance surveillance recommendations [27,28]. MRSA remains a major cause of complicated skin and respiratory infections in children and is increasingly identified outside hospital settings.

In Kosovo, the absence of a national pediatric antimicrobial resistance surveillance system limits the ability to directly correlate prescribing patterns with resistance trends. Regional data and World Health Organization reports indicate that countries with high outpatient antibiotic consumption are disproportionately affected by antimicrobial resistance [26]. The elevated use of macrolides and cephalosporins in Ferizaj observed in the present study may therefore reflect an increased risk for resistance emergence if corrective stewardship measures are not implemented.

These findings highlight the urgent need for national pediatric antimicrobial stewardship programs, integration of microbiological diagnostics into primary care, and continuous monitoring of antibiotic consumption patterns as key components for mitigating the spread of multidrug resistance in Kosovo’s pediatric population.

The pandemic further exacerbated these patterns by reducing access to laboratory diagnostics, a challenge that remains evident in Kosovo even in the post-COVID period. Expanding point-of-care diagnostics such as CRP testing and rapid streptococcal antigen detection—recommended by WHO and EU antimicrobial stewardship programs—would likely reduce unnecessary prescribing [8].

Seasonal variation was pronounced, with winter showing the highest prevalence of antibiotic use. This mirrors the late-pandemic rebound observed in EU/EEA countries, where antibiotic consumption increased markedly during the winter seasons of 2022–2023 following an earlier pandemic-related decline [29]. Similar seasonal peaks have been reported in Sweden, the United States, and other European settings [17,18].

Geographical differences were also notable. Ferizaj consistently demonstrated higher prescribing rates and greater reliance on broad-spectrum antibiotics compared with Prishtina. Comparable regional variations have been documented in studies from Italy and Spain, where differences in physician experience, diagnostic availability, and parental pressure contribute to variable antibiotic use [10,19]. The absence of national pediatric prescribing guidelines in Kosovo likely amplifies these discrepancies.

Overall, while some patterns observed in Kosovo—such as the predominance of penicillins, higher use in toddlers, and strong winter seasonality—align with global findings, the high use of macrolides and cephalosporins, relatively low guideline adherence (61.4%), and marked regional variation differ substantially from Western European benchmarks, where guideline adherence generally exceeds 75% [20]. These findings underscore the urgent need for national pediatric prescribing guidelines, expansion of diagnostic tools, and structured stewardship interventions to optimize antibiotic use and mitigate antimicrobial resistance risks. Additional details on infectious diagnoses and inappropriate antibiotic prescribing by age group are provided in Supplementary Table S1.

## 4. Methodology

Study design and setting: This retrospective, observational, and quantitative study was conducted in primary health care (PHC) centers in the municipalities of Prishtina and Ferizaj, Kosovo. The study period covered January 2022 to December 2025, representing the post-COVID-19 timeframe during which empirical antibiotic use remained common due to limited access to diagnostic tools.

Prishtina, the capital city, has a larger pediatric population and greater availability of pediatricians and diagnostic services. Ferizaj, while the second-largest municipality in the study, relies more heavily on general practitioners and has more limited point-of-care diagnostic capacity. CRP rapid testing and streptococcal antigen tests were intermittently available in Prishtina but were rarely accessible in Ferizaj, contributing to reliance on symptom-based empirical prescribing.

Study population and inclusion criteria

All medical records of children aged 1–7 years who visited participating PHC centers during the study period were screened. Records were included if they met the following criteria:

- Complete diagnostic information;
- Documented antibiotic prescription status;
- Patient age between 1 and 7 years.

Records with missing diagnostic data, incomplete prescription fields, duplicated entries, or age outside the target range were excluded. Missing or inconsistent entries were resolved by cross-checking with protocol books. Where discrepancies could not be resolved, cases were excluded to avoid misclassification bias.

Data sources and collection

Data were extracted from the national electronic health record (EHR) system and verified using protocol books maintained by each PHC center to ensure completeness and accuracy. Variables collected included age, sex, municipality, season of visit, diagnosis, antibiotic class and agent, dosage form, route of administration, and adherence to international pediatric prescribing guidelines.

A standardized data extraction tool, adapted from the European Surveillance of Antimicrobial Consumption (ESAC) and WHO Point Prevalence Survey frameworks, was used to minimize variability and reduce information bias. Two independent researchers checked all entries for accuracy.

Assessment of prescription appropriateness

Appropriateness of antibiotic prescriptions was assessed independently by two senior physicians (a pediatrician and an infectious disease specialist) and a clinical pharmacist, based on internationally recognized pediatric guidelines, including:

- WHO Model List of Essential Medicines for Children (2021);
- ESPID (European Society for Paediatric Infectious Diseases) guidelines;
- NICE (National Institute for Health and Care Excellence) pediatric infection guidelines.
  Prescriptions were categorized as follows:
- Appropriate: correct indication, agent, dose, and duration.
- Partially appropriate: correct indication but suboptimal agent, dose, or duration.
- Inappropriate: incorrect indication, unjustified broad-spectrum use, or deviation from recommended first-line therapy.

Disagreements were resolved by consensus. Inter-rater reliability was high (κ = 0.82), indicating strong agreement among evaluators.

Statistical analysis

Descriptive statistics (frequencies, percentages, means ± SD) summarized demographic and prescribing characteristics. Differences between Prishtina and Ferizaj were evaluated using Chi-square tests for categorical variables and Student’s *t*-test for continuous variables. Seasonal and annual prescribing trends were assessed using the Cochran–Armitage trend test.

Multivariable logistic regression was performed to identify independent predictors of antibiotic prescribing, including age group, season, municipality, and sex. Results were expressed as odds ratios (ORs) with 95% confidence intervals (CIs). A *p*-value < 0.05 was considered statistically significant. Model assumptions (linearity, multicollinearity, residual analysis) were checked prior to analysis.

Ethical considerations

Ethical approval was obtained from the Ethics Committee of the Faculty of Medical Sciences at UBT College. Additional authorization was granted by municipal health authorities in Prishtina and Ferizaj (10 January 2022). As all data were anonymized and retrospectively extracted from EHRs and protocol books, informed consent was waived. The study adhered to the ethical standards of the Declaration of Helsinki (2013).

Study Limitations

This study has several limitations that should be considered when interpreting the findings. First, the retrospective observational design limited access to clinical details underlying prescribing decisions, such as physicians’ diagnostic reasoning and parental pressure. As a result, causal inferences could not be established between clinical presentations and antibiotic prescribing behavior.

Second, microbiological data were unavailable, and antibiotic prescriptions could not be directly linked to culture results or pathogen susceptibility patterns. Consequently, resistance trends could not be assessed, nor could the appropriateness of antibiotic selection be evaluated against confirmed microbiological diagnoses.

In addition, vulnerability-related clinical variables such as immunodeficiency status, prematurity, and chronic conditions were not systematically captured in the health records, preventing stratified analysis of high-risk pediatric subgroups.

Finally, the study was conducted in two major municipalities, which may limit generalizability to other regions of Kosovo with different health care infrastructures and prescribing behaviors.

Despite these limitations, the large sample size and real-world data obtained from the national EHR system provide a robust overview of pediatric antibiotic prescribing practices in Kosovo and allow meaningful comparisons between regions. Descriptive and inferential statistical analyses were conducted as appropriate. All statistical analyses were performed using IBM SPSS Statistics for Windows, Version 26.0 (IBM Corp., Armonk, NY, USA).

## 5. Conclusions

This study, conducted in two major municipalities of Kosovo between January 2022 and December 2025, demonstrated that antibiotic prescribing among children aged 1–7 years remains high, with an overall prevalence of 30.7%. Significant inter-municipality differences were observed, with Ferizaj showing consistently higher prescribing rates and a greater reliance on broad-spectrum antibiotics, while penicillins predominated in Prishtina. Younger age (1–3 years), winter season, and local prescribing habits emerged as the strongest predictors of antibiotic use.

A key finding was that only 61.4% of prescriptions adhered fully to international pediatric guidelines, indicating substantial inappropriate or partially appropriate prescribing. These results highlight the urgent need for structured antibiotic stewardship interventions in primary care settings in Kosovo. Priority actions should include the development of standardized pediatric prescribing protocols, continuous professional education for primary-care clinicians, targeted parental awareness campaigns, and systematic monitoring of pediatric antibiotic use through routine audit and feedback mechanisms.

Consistent with trends reported across Europe and the United States, our findings reflect broader seasonal and demographic patterns while also revealing unique challenges in the Kosovar context—particularly the elevated use of macrolides and cephalosporins in Ferizaj. Implementing evidence-based policies, strengthening diagnostic support, and reinforcing stewardship efforts are essential steps to prevent further escalation of antimicrobial resistance and to ensure safe, effective, and guideline-concordant antibiotic use for children in Kosovo.

## Figures and Tables

**Table 1:** Prevalence of antibiotic prescriptions by municipality.

Municipality	Total Visits	With Antibiotic (*n*)	Prevalence (%)
Pristina	2210	630	28.5
Ferizaj	2110	698	34.2
Total	4320	1328	30.7

**Table 2:** Distribution of antibiotics by class and municipality.

Class	Pristina *n* (%)	Ferizaj *n* (%)	Total *n* (%)
Amoxicillin–Clavulanic Acid	270 (42.8)	300 (43.0)	570 (42.9)
Amoxicillin	135 (21.5)	150 (21.5)	285 (21.5)
Metronidazole	103 (16.3)	115 (16.5)	218 (16.4)
Macrolides	39 (6.2)	82 (11.7)	121 (9.1)
Cephalosporins	21 (3.4)	55 (7.9)	76 (5.7)
Others	62 (9.8)	56 (8.0)	118 (8.9)

**Table 3:** Summarizes the appropriateness of antibiotic prescriptions based on international pediatric guidelines.

Category	Number (*n*)	Percentage (%)
Appropriate	815	61.4
Partially appropriate	280	21.1
Inappropriate	233	17.5
Total	1328	100.0

**Table 4:** Antibiotic prescribing prevalence by age group.

Age Group	Total Visits	With Antibiotic (*n*)	Prevalence (%)
1–3 years	1900	667	35.1
4–7 years	2420	661	27.3
Total	4320	1328	30.7

**Table 5:** Seasonal variation in antibiotic prescribing.

**Season**	**Total Visits**	**With Antibiotic (*n*)**	**Prevalence (%)**
Winter	1200	467	38.9
Spring	1050	309	29.4
Summer	980	220	22.5
Autumn	1090	332	30.5
Total	4320	1328	30.7

**Table 6:** Independent predictors of antibiotic prescribing identified through multivariable logistic regression.

Factor	OR	95% CI	*p*-Value
Age 1–3 (vs. 4–7)	1.42	1.21–1.66	<0.001
Winter (vs. Summer)	1.85	1.55–2.20	<0.001
Ferizaj (vs. Pristina)	1.34	1.12–1.59	0.001
Male (vs. Female)	1.08	0.92–1.27	0.35

**Table 7:** Predictors associated with macrolide prescribing.

Factor	OR	95% CI	*p*-Value
Ferizaj vs. Pristina	1.55	1.22–1.97	<0.001
Winter vs. Summer	1.6	1.25–2.05	<0.001
Age 1–3 vs. 4–7	1.1	0.89–1.35	0.40

**Table 8:** Yearly trends in antibiotic prescribing prevalence.

Year	Total Visits	With Antibiotic (*n*)	Prevalence (%)
2022	1000	290	29.0
2023	1050	315	30.0
2024	1150	360	31.3
2025	1120	363	32.4

**Table 9:** Presents annual antibiotic prescribing prevalence stratified by municipality.

Year	Pristina (%)	Ferizaj (%)
2022	27.0	31.2
2023	28.5	32.0
2024	29.0	34.0
2025	29.6	35.1

## Data Availability

The data presented in this study are available from the corresponding author upon reasonable request. The data are not publicly available due to ethical and privacy restrictions.

## Supplementary Materials

The following supporting information can be downloaded at: https://www.mdpi.com/article/10.3390/antibiotics14121282/s1.
