# Supplementary material for: Prevalence and Patterns of Antibiotic Prescribing Among Children Aged 1–7 Years in Primary Health Care Centers in Prishtina and Ferizaj, Kosovo (2022–2025): A Retrospective Observational Study
Source: Antibiotics (Basel). 2025 Dec 18;14(12):1282. doi: 10.3390/antibiotics14121282 (PMC12729371; doi:10.3390/antibiotics14121282)
Supplement: Supplementary file 1 [file antibiotics-14-01282-s001.zip › antibiotics-3949471-supplementary.pdf]

Note: Data for January 2025 were excluded to avoid misinterpretation, as they represent only one month.
Note: Data for January 2025 were excluded to avoid misinterpretation, as they represent only one month.

Supplementary Table S1: Supplement S1 (Diagnosis vs Antibiotic Class)

The following table presents the most frequent infectious diagnoses and the proportion of inappropriate prescriptions by age group.

Diagnosis	Total Cases (n)	Inappropriate Prescriptions (n)	Inappropriate %
Respiratory tract infections	600	180	30.0%
Otitis media	250	50	20.0%
Urinary tract infections	120	25	20.8%
Gastrointestinal infections	100	20	20.0%

Figure S1: Trend of Prescribing Rates

Reformatted visualization of yearly prescribing rates, excluding January 2025 data.

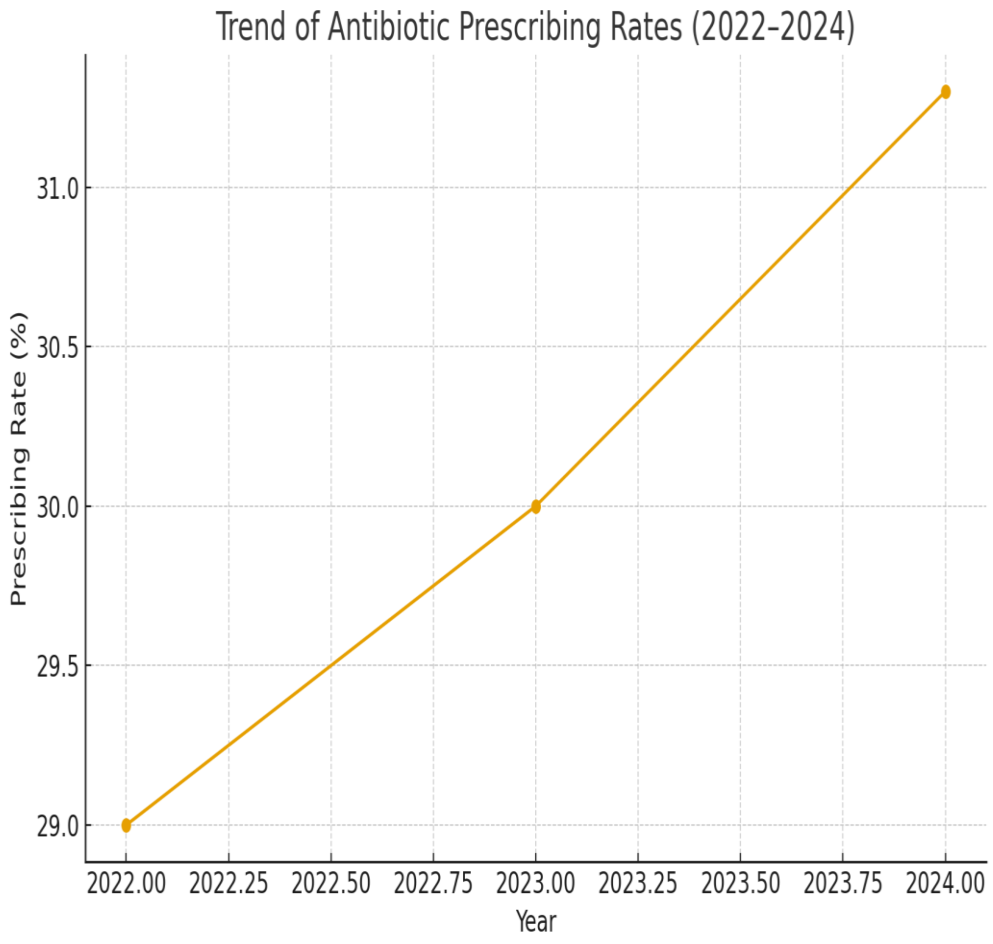

Antibiotic Resistance — Quantitative Context

We integrated quantitative data from ECDC/CAESAR and regional surveillance reports, noting resistance rates in Streptococcus pneumoniae to macrolides (>20% in neighboring countries) and Enterobacterales to third-generation cephalosporins (>15%). Kosovo lacks national pediatric-specific data, which we clearly acknowledge, but these proxies highlight the importance of stewardship.

Drivers of Macrolide and Cephalosporin Increase

The observed increase in macrolide and cephalosporin prescriptions likely stems from perceived broad-spectrum coverage, seasonal respiratory infection surges, market availability, and empirical substitution when resistance to penicillins is suspected.

Practical Guidance for Empirical Prescribing in Kosovo

Practical recommendations for pediatric primary care include: Amoxicillin as first-line for URTI and otitis media; macrolides restricted to penicillin allergy or suspected atypical pathogens; cephalosporins reserved for clinically justified spectrum expansion; metronidazole for anaerobic/GI infections; and delayed-prescription strategies with parental counseling.

Role of Clinical Pharmacists and ID Specialists

Clinical pharmacists should systematically review prescriptions for dose, duration, and appropriateness, while ID fellows or specialists may support primary care through regional tele-consultation mechanisms.

Role of Microbiology Laboratories

Microbiology laboratories play a crucial role in UTI diagnosis through culture/susceptibility, and should be expanded for point-of-care tests (CRP, throat swabs). Strengthened lab feedback loops will enable targeted therapy and de-escalation of broad-spectrum use.

Limitations (Expanded)

Our retrospective design limited access to clinicians' rationale behind prescribing. Kosovo currently lacks national pediatric AMR surveillance data, and laboratory use was limited in primary care. These factors restrict generalizability but underscore the urgency for standardized pediatric guidelines and monitoring systems.
